# Analytical Study of Front-End Circuits Coupled to Silicon Photomultipliers for Timing Performance Estimation under the Influence of Parasitic Components

**DOI:** 10.3390/s20164428

**Published:** 2020-08-08

**Authors:** Pietro Antonio Paolo Calò, Savino Petrignani, Michele Di Gioia, Cristoforo Marzocca

**Affiliations:** Department of Electrical and Information Engineering, Politecnico di Bari, via E. Orabona 4, I-70125 Bari, Italy; savino.petrignani@poliba.it (S.P.); m.digioia2@studenti.poliba.it (M.D.G.)

**Keywords:** silicon photomultiplier, front-end electronics, single-photon response, timing accuracy

## Abstract

Full exploitation of the intrinsic fast timing capabilities of analog silicon photomultipliers (SiPMs) requires suitable front-end electronics. Even a parasitic inductance of a few nH, associated to the interconnections between the SiPM and the preamplifier, can significantly degrade the steepness of the detector response, thus compromising the timing accuracy. In this work, we propose a simple analytic expression for the single-photon response of a SiPM coupled to the front-end electronics, as a function of the main parameters of the detector and the preamplifier, taking into account the parasitic inductance. The model is useful to evaluate the influence of each parameter of the system on the slope of its response and to guide the designer in the definition of the architecture and the specifications for the front-end electronics. The results provided by the model have been successfully compared with experimental measurements from a front-end circuit with variable configuration based on a bipolar junction transistor (BJT), coupled to a 3 × 3 mm^2^ SiPM stimulated by a fast-pulsed laser source.

## 1. Introduction

Accuracy in time measurements represents a challenging task for a growing number of photo-detection systems in applications such as time-of-flight positron emission tomography (ToF-PET) [1], γ-ray spectroscopy [2], time-correlated single photon counting (TCSPC) [3,4], and distance measurements (LiDAR) [5,6]. In this kind of applications, time resolution as low as 100 ps FWHM, or even less, are often required.

Silicon photomultipliers (SiPMs) are becoming the detectors of choice for such applications, owing to their intrinsically fast response, characterized by sub-nanosecond risetime, and single-photon sensitivity. The design of effective SiPM-based detection systems aiming at good single-photon time resolution calls for the development of high-speed and low-noise front-end electronics. When the detector equivalent capacitance is quite large, as in the case of SiPMs, the best achievable time resolution is often constrained by the characteristics of the front-end electronics [7]. Indeed, electronic noise is one of the main causes of jitter in time measurements and often dominates other sources of error, such as the intrinsic jitter due to avalanche build-up statistics [8,9]. The effects of electronic noise are emphasized when the maximum slope of the output pulse produced by the front-end electronics is reduced because of either non-optimal coupling between detector and preamplifier or bandwidth limitations.

In fact, the most common time pick-off technique is leading-edge discrimination. A fast comparator, cascaded to the preamplifier, fires when the signal overcomes a suitable threshold V_TH_. The resulting rms time jitter $\sigma_{t}$ is expressed by the following well-known equation:

(1) $$\sigma_{t}=\frac{\sigma_{n}}{{\frac{{\mathrm{dV}}_{\mathrm{out}}\left(t\right)}{\mathrm{dt}}|}_{V_{\mathrm{out}}=V_{\mathrm{TH}}}},$$

where $\sigma_{n}$ is the *rms* electronic noise at the preamplifier output, and the denominator represents the slope of the output pulse V_out_(t), evaluated around the level of the discriminator threshold [10]. Hence, whenever good timing accuracy is required, it is important to figure out how the parameters of the front-end electronics, such as the input resistance and the bandwidth, affect the leading edge of the output pulse and its slope.

For this purpose, even though powerful simulation tools and accurate models of the system can be used, analytical expressions of the response V_out_(t) and its slope as a function of the main parameters involved can be very helpful. For instance, if these expressions are available, the initial choice of the specifications for the front-end electronics can be easily made. Moreover, the designer can understand with little effort in which direction the circuit must be modified to optimize the time performance of the detection system. From this perspective, useful analytical expressions of V_out_(t) are already available in the literature [11,12,13], but their accuracy can be improved if relevant parameters, not yet considered, are added to the model used.

In fact, it is well known that also the small parasitic inductance L_par_, associated to the interconnection between the SiPM and the front-end electronics, plays a relevant role in shaping the very fast waveform of the SiPM signal [14,15]. Consequently, an effective model of the whole detection system, besides including the parameters of both the SiPM and the preamplifier, must also take account of this inductance. This results in increased complexity of the transfer function of the system, which makes it very difficult to obtain the desired analytical expression for both the system response and its slope as a function of time. Previous attempts have been made in this regard, but they have been based on an oversimplified detector model and are not supported by experimental validation [16].

We propose a new approximate analysis which results in few simple mathematical equations that relate some features of the single-photon time response of a SiPM readout circuit to the most important circuit parameters, including the parasitic inductance L_par_. In particular, the approximate expression obtained for the slope of the single-photon response correctly reproduces the behavior of the complete model of the system.

A factorized expression of the overall system transfer function is derived, such that the individual contributions of the parameters involved to the formation of the leading edge of the output response can be easily identified and evaluated.

The proposed analysis allows us to draw some conclusions about the way parasitic components interact with the detector and circuit parameters and influence the timing performance of a SiPM-based detection system. The resulting analytic expressions provide the designer of the front-end with practical guidelines for the selection of the most suitable architecture and useful indications about its specifications.

Experiments carried out with a 3 × 3 mm^2^ SiPM coupled to a BJT-based front-end circuit have also been carried out to validate the effectiveness of the proposed analysis. The achieved results show that our analytical model reproduces the slope of the single-photon circuit response as a function of the main parameters involved.

## 2. Transfer Function of a Typical SiPM-Based Detection System

**Figure 1:**
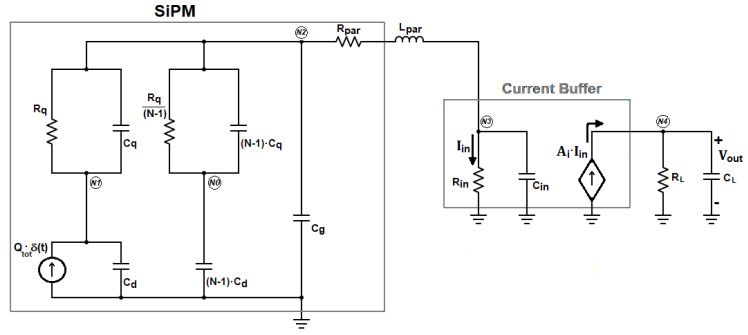
Equivalent electrical model of the silicon photomultiplier (SiPM) coupled to a current-mode front-end.

The complete electrical model of a SiPM detector with N micro-cells coupled to a front-end circuit based on the classic current-mode approach [17,18] is shown in Figure 1. The preamplifier is a current buffer with gain A_i_, which exhibits a low input resistance R_in_. The load resistance R_L_ converts the output current of the buffer into the voltage V_out_, whereas the bandwidth of the preamplifier is dominated by the single output time constant τ_L_ = R_L_C_L_.

In Figure 1, only one micro-cell of the detector is supposed to undergo a Geiger discharge, since our analysis is restricted to single-photon events.

The model of the SiPM includes the delta-like current source I_μ__cell_(t) = Q_tot_∙δ(t), which represents the very fast Geiger discharge of the fired micro-cell, the quenching resistor R_q_, the parasitic capacitance across it C_q_, the photodiode capacitance C_d_, and the capacitance C_g_, due to the routing metal grid used to connect in parallel all the micro-cells. L_par_ represents the parasitic inductance associated with the interconnection wire between the SiPM and the front-end.

**Figure 2:**
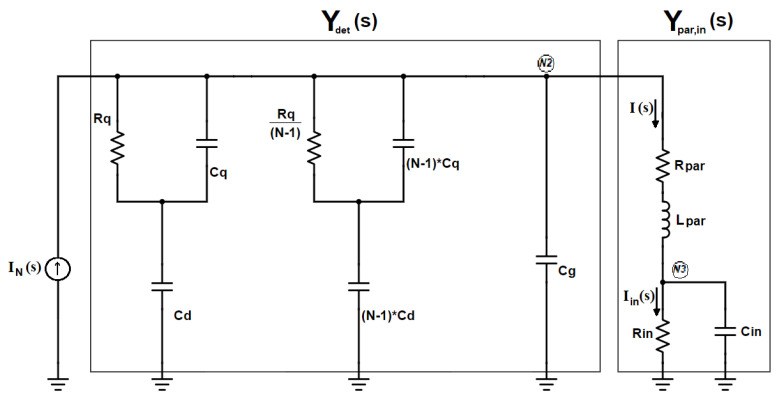
The input section of the circuit in Figure 1, redrawn as the parallel of two admittances, Y_det_ and Y_par,in_.

The same electrical model of the SiPM has been already used to derive the transfer function from the input Dirac’s delta to the current I_in_ flowing through R_in_ in Figure 1 [19,20,21]. Compared with the analysis proposed in Ciciriello et al. [19], limited to the Laplace domain, the only additional element introduced in the model is the resistor R_par_. This component, as described in Licciulli et al. [22], includes both the series substrate resistance of the SiPM, R_sub_, that improves the accuracy of the SiPM model, and other series parasitic resistances associated with the interconnections between the detector and the electronics. In all the practical cases the contribution of R_sub_ is dominant in R_par,_ thus R_sub_ ≅ R_par_. To simplify the analysis in the s-domain, the input section of the circuit in Figure 1 has been redrawn, after applying the Norton equivalent to the SiPM model, resulting in the schematic of Figure 2.

The expression of the Norton equivalent current I_N_(s) in Figure 2 is the following:

(2) $$I_{N}\left(s\right)=Q_{\mathrm{tot}}\frac{1+\tau_{q}s}{1+\tau_{r}s},$$

where the time constants $\tau_{q}=R_{q}C_{q}$ and $\tau_{r}=R_{q}\left(C_{d}+C_{q}\right)$ appear.

After expressing the parallel admittances $Y_{\det}\left(s\right)$ and $Y_{\mathrm{par},\mathrm{in}}\left(s\right)$ respectively as

(3) $$Y_{\det}\left(s\right)={\mathrm{NC}}_{d}\frac{s\;\left(1+\tau_{q}s\right)}{1+\tau_{r}s}+{\mathrm{sC}}_{g},$$

(4) $$Y_{\mathrm{par},\mathrm{in}}\left(s\right)=\frac{1}{R_{s}}\cdot \frac{\omega_{n}^{2}}{s^{2}+2\;\zeta \;\omega_{n}\;s+\omega_{n}^{2}}\;\left(1+\tau_{\mathrm{in}}s\right),$$

where $\tau_{\mathrm{in}}=R_{\mathrm{in}}C_{\mathrm{in}}$, $R_{s}=R_{\mathrm{in}}+R_{\mathrm{par}}$, $\omega_{n}^{2}=\frac{1}{L_{\mathrm{par}}C_{\mathrm{in}}}\cdot \frac{R_{s}}{R_{\mathrm{in}}}=\frac{1}{\tau_{\mathrm{in}}}\cdot \frac{R_{s}}{L_{\mathrm{par}}}$, and $2\;\zeta \;\omega_{n}=\frac{1}{\tau_{\mathrm{in}}}+\frac{R_{\mathrm{par}}}{L_{\mathrm{par}}}$, application of the current divider rule leads to the following expression for the current I_in_(s):

(5) $$I_{\mathrm{in}}\left(s\right)=\frac{1}{1+\tau_{\mathrm{in}}s}\cdot \frac{Y_{\mathrm{par},\mathrm{in}}\left(s\right)}{Y_{\mathrm{par},\mathrm{in}}\left(s\right)+Y_{\det}\left(s\right)}\cdot I_{N}\left(s\right).$$

Replacing expressions from (2) to (4) in (5), the Laplace transform of the current flowing into the input resistance of the preamplifier R_in_ can be rearranged as follows:

(6) $$I_{\mathrm{in}}\left(s\right)=\frac{Q_{\mathrm{tot}}\omega_{n}^{2}\left(1+\tau_{q}s\right)}{\omega_{n}^{2}\left(1+\tau_{\mathrm{in}}s\right)\left(1+\tau_{r}s\right)+R_{s}s\left(s^{2}+2\;\zeta \;\omega_{n}\;s+\omega_{n}^{2}\right)\left[C_{g}\left(1+\tau_{r}s\right)+{\mathrm{NC}}_{d}\left(1+\tau_{q}s\right)\right]}$$

In Figure 1, the output current of the current buffer is converted into the output voltage

(7) $$V_{\mathrm{out}}\left(s\right)=\frac{K_{R}}{\left(1+\tau_{L}s\right)}\cdot I_{\mathrm{in}}\left(s\right),$$

where K_R_ = A_i_·R_L_ represents the overall transimpedance gain of the preamplifier.

Equations (6) and (7) can be expressed in the following way:

(8) $$I_{\mathrm{in}}\left(s\right)=Q_{\mathrm{tot}}\cdot \left(\frac{1+\tau_{q}s}{1+a_{1}s+a_{2}s^{2}+a_{3}s^{3}+a_{4}s^{4}}\right),$$

(9) $$V_{\mathrm{out}}\left(s\right)=Q_{\mathrm{tot}}\cdot K_{R}\cdot \left[\frac{1+\tau_{q}s}{\left(1+\tau_{L}s\right)\left(1+a_{1}s+a_{2}s^{2}+a_{3}s^{3}+a_{4}s^{4}\right)}\right],$$

where

$$\begin{array}{l} a_{1}=\tau_{\mathrm{in}}+\tau_{r}+R_{s}\left(C_{g}+{\mathrm{NC}}_{d}\right),\\ a_{2}=\tau_{\mathrm{in}}\tau_{r}+C_{g}\left(R_{s}\tau_{r}+L_{\mathrm{par}}+R_{\mathrm{par}}\tau_{\mathrm{in}}\right)+{\mathrm{NC}}_{d}\left(R_{s}\tau_{q}+L_{\mathrm{par}}+R_{\mathrm{par}}\tau_{\mathrm{in}}\right),\\ a_{3}=L_{\mathrm{par}}\left(C_{g}+{\mathrm{NC}}_{d}\right)\tau_{\mathrm{in}}+\left(L_{\mathrm{par}}+R_{\mathrm{par}}\tau_{\mathrm{in}}\right)\left(C_{g}\tau_{r}+{\mathrm{NC}}_{d}\tau_{q}\right),\\ a_{4}=L_{\mathrm{par}}(C_{g}\tau_{r}+{\mathrm{NC}}_{d}\tau_{q})\tau_{\mathrm{in}}. \end{array}$$

Equation (9) is the relation between the total charge Q_tot_ released by a single fired micro-cell and the voltage at the output of the current-mode preamplifier in Figure 1.

**Figure 3:**
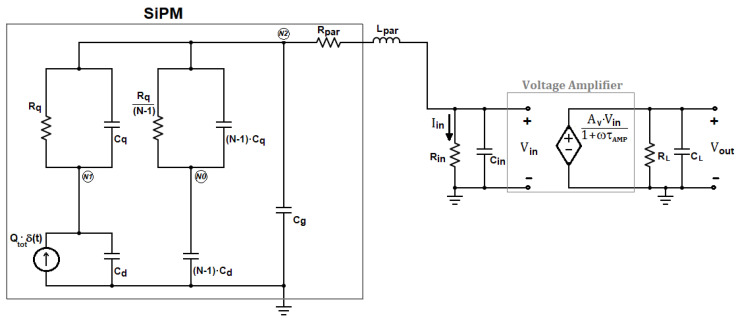
Model of the SiPM coupled to a voltage-mode front-end circuit.

In case a voltage-mode approach is used for the front-end, as, for instance, in Di Lorenzo et al. [23] and Fisher et al. [24], the current pulse of the detector is first converted into a voltage by means of the input resistor R_in_. The voltage across R_in_ is amplified by means of a voltage amplifier with gain A_V_, as depicted in Figure 3.

In this case, the output voltage V_out_(s) can still be expressed by Equation (9). The only formal difference is that the overall transimpedance gain K_R_ = A_i_·R_L_ of the current-mode approach must be replaced with the factor K_R_’ = A_V_·R_in_, and the dominant time constant of the voltage amplifier τ_AMP_ must be considered instead of τ_L_.

The transfer function in Equation (9) is characterized by one zero and five poles, thus the corresponding expression of the pulse I_in_(t) in the time-domain is rather complex. This expression can be either calculated as the inverse Laplace transform of Equation (9), for instance using a MATLAB^®^ script, or plotted by means of SPICE simulations of the circuit in Figure 1. In both cases, to distinguish the influence of each model parameter on the slope of the leading edge of the pulse, sets of parametric simulations would be required. In order to effectively support the choice of the architecture and the design of the preamplifier, it is more convenient to simplify the complex analytic expressions derived so far, considering suitable approximations.

## 3. Analytic Approximation of the Model for the Study of the Fast Transient

We are interested in the fast initial transient of the output pulse, which dominates the timing performance of the detection system. Thus, an analytic approximation of the model that does not affect the high frequency components of the response is needed. In this perspective, the slow second order term $\left(1+\tau_{\mathrm{in}}s\right)\left(1+\tau_{r}s\right)$ can be neglected in Equation (6), since its contribution is irrelevant as compared to the remaining fourth order polynomial of the denominator.

The resulting expression of the current I_in_(s), valid for the early fast transient of the response, can be written and factorized as follows:

(10) $$I_{\mathrm{in}}\left(s\right)≅V_{0}\cdot \frac{\left(1+\tau_{q}s\right)}{s\left(1+\tau_{p}s\right)}\cdot \frac{1}{R_{s}}\cdot \frac{\omega_{n}^{2}}{\left(s^{2}+2\;\zeta \;\omega_{n}\;s+\omega_{n}^{2}\right)},$$

where $\tau_{p}=\frac{C_{g}\tau_{r}+{\mathrm{NC}}_{d}\tau_{q}}{C_{g}+{\mathrm{NC}}_{d}}\;\mathrm{and}\;V_{0}=\frac{Q_{\mathrm{tot}}}{C_{g}+{\mathrm{NC}}_{d}}$.

By substituting expression (10) in (7), the following approximation for the output pulse $V_{\mathrm{out}}\left(s\right)$ is obtained:

(11) $$V_{\mathrm{out}}\left(s\right)≅V_{0}\cdot \frac{\left(1+\tau_{q}s\right)}{s\left(1+\tau_{p}s\right)}\cdot \frac{1}{R_{s}}\cdot \frac{\omega_{n}^{2}}{\left(s^{2}+2\;\zeta \;\omega_{n}\;s+\omega_{n}^{2}\right)}\cdot \frac{K_{R}}{\left(1+\tau_{L}s\right)}.$$

**Figure 4:**
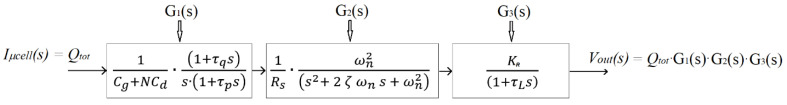
Simplified block diagram of the system.

In Equation (11), the whole system is represented in the s-domain as the cascade of three submodules, as shown in Figure 4. The blocks of Figure 4 correspond to the factors of Equation (11). $G_{1}\left(s\right)$ accounts for the SiPM model with its electrical parameters; $G_{2}\left(s\right)$ models the interaction between the SiPM and the preamplifier, i.e., the interconnections and the input impedance of the current buffer; $G_{3}\left(s\right)$ represents the current-to-voltage transfer function of the preamplifier.

Thus, expression (11) allows isolating the contribution of each of the main blocks of the detection chain to the shaping of the initial current pulse.

The results provided by expressions (9) and (11) have been compared, using the set of electrical parameters of the SiPM S10931-050P from Hamamatsu reported in Table 1, extracted by applying the procedure described in Licciulli [22].

**Figure 5:**
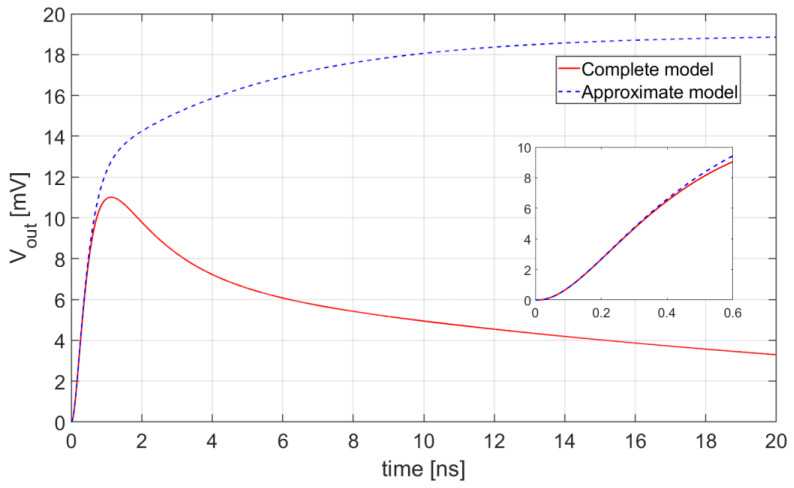
Comparison between the waveforms V_out_(t) obtained from Equations (9) (complete model) and (11) (approximate model). The inset shows the early part of the transient response.

The inverse Laplace transforms of Expressions (9) and (11) are plotted in Figure 5. They represent, respectively, the response of the complete model and the response of its approximation, valid for high frequencies, when just one micro-cell of the SiPM undergoes avalanche breakdown with the following set of parameters: L_par_ = 10 nH, R_in_ = 10 Ω, C_in_ = 1 pF, K_R_ = 1.2 kΩ, and BW = 1/2πτ_L_ = 1 GHz.

The inset in Figure 5 proves that the approximation of $V_{\mathrm{out}}\left(t\right)$ and the complete model fit almost perfectly in the region of interest for time pickoff, with only marginal deviations. The two expressions start to diverge only in proximity of the peak of the exact model. It is worth noting that good fittings are obtained regardless of any realistic choice of the parameter values.

As already mentioned, the slope of the response at the chosen threshold V_TH_ is a key parameter for time resolution: once established the noise level, the steeper the waveform at the threshold crossing point, the lower the jitter.

**Figure 6:**
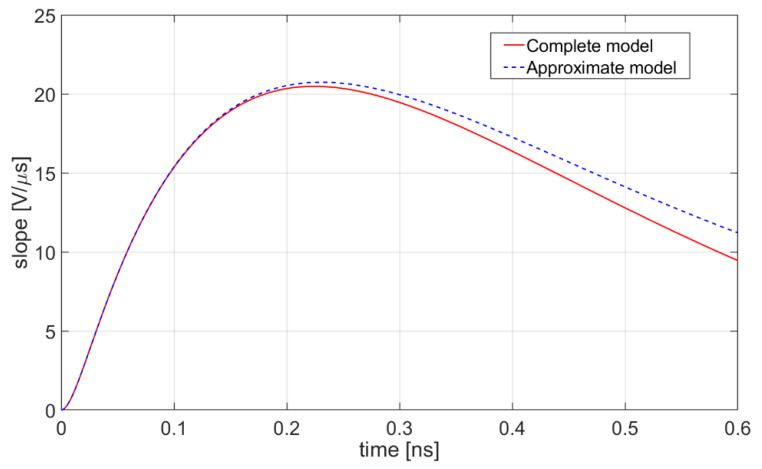
Time derivatives of the response V_out_ (t): comparison between the complete model in Equation (9) and its approximation in Equation (11).

Figure 6 shows the time derivatives of the waveforms in Figure 5 in the portion of their rising edge, confirming the validity of approximation (11).

**Figure 7:**
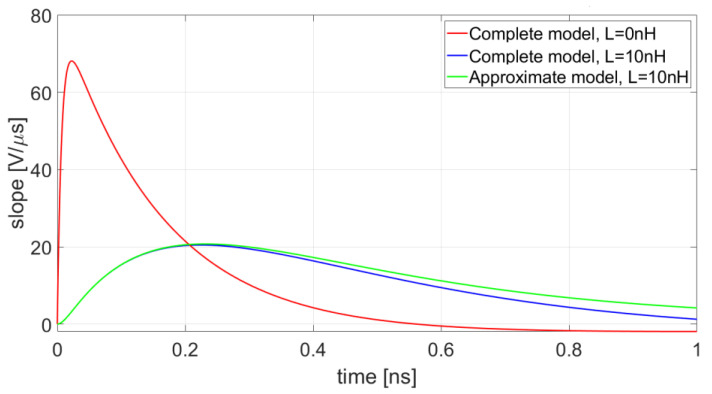
Slope of output pulses obtained with no series inductance and with a 10 nH inductance included in both the complete and approximate models.

In general, the slope of the response exhibits a strong dependence on the value of the series inductance. Figure 7 shows that also a small 10 nH inductance remarkably affects the shape and the amplitude of the slope of the response. It is also apparent that the approximate model can predict the slope of the pulse even in presence of such a small inductance.

## 4. A Comprehensive Analysis Including the Front-End Bandwidth

To account for the effect of the finite bandwidth of the front-end upon the shape of the response and its slope, in Equation (11), the preamplifier has been described as a single pole transfer function, as reported hereinafter for convenience:

(12) $$V_{I,\;\mathrm{out}}\left(s\right)≅V_{0}\cdot \frac{\left(1+\tau_{q}s\right)}{s\left(1+\tau_{p}s\right)}\cdot \frac{\omega_{n}^{2}}{R_{s}\left(s^{2}+2\;\zeta \;\omega_{n}\;s+\omega_{n}^{2}\right)}\cdot \frac{K_{R}}{\left(1+\tau_{L}s\right)}.$$

In Equation (12), the subscript “I” has been added to emphasize that we are referring to a current-mode approach. Here, a transimpedance preamplifier with gain K_R_ = A_i_·R_L_ and cut-off frequency set by the dominant time constant $\tau_{L}$ is used to convert the current pulse $I_{\mathrm{in}}\left(t\right)$ into the output voltage $V_{I,\mathrm{out}}\left(t\right)$, as illustrated in Figure 1. As already pointed out, in case a voltage-mode readout is adopted, (see Figure 3)**,** the gain K_R_ is replaced with the product K_R_’ = A_v_·R_in_ and the time constant τ_L_ is replaced with τ_AMP_, resulting in the following expression of the output voltage pulse:

(13) $$V_{V,\;\mathrm{out}}\left(s\right)≅V_{0}\cdot \frac{\left(1+\tau_{q}s\right)}{s\left(1+\tau_{p}s\right)}\cdot \frac{\omega_{n}^{2}}{R_{s}\left(s^{2}+2\;\zeta \;\omega_{n}\;s+\omega_{n}^{2}\right)}\cdot \frac{A_{v}\cdot R_{\mathrm{in}}}{\left(1+\tau_{\mathrm{AMP}}s\right)}$$

By observing that, in practical cases, $\tau_{\mathrm{in}}=R_{\mathrm{in}}C_{\mathrm{in}}\ll \frac{L_{\mathrm{par}}}{R_{\mathrm{par}}}$, the term $2\;\zeta \;\omega_{n}$ can be approximated to $\frac{1}{\tau_{\mathrm{in}}}$. Thus, the second order contribution that appears in Equations (12) and (13) can be further simplified as follows:

(14) $$G_{2}\left(s\right)=\frac{\omega_{n}^{2}}{R_{s}\left(s^{2}+2\;\zeta \;\omega_{n}\;s+\omega_{n}^{2}\right)}≅\frac{1}{R_{s}}\frac{\frac{1}{\tau_{\mathrm{in}}\cdot \tau_{A}}}{\left(s^{2}+\frac{s}{\tau_{\mathrm{in}}}+\frac{1}{\tau_{\mathrm{in}}\cdot \tau_{A}}\right)},$$

where $\tau_{A}=\frac{L_{\mathrm{par}}}{R_{s}}$ and the two poles of the system are respectively equal to −1/τ_A_ and −1/τ_in_.

The time constant τ_in_ is very small compared with τ_A_ (typically τ_A_/τ_in_ > 10) and, consequently, the faster exponential term associated with τ_in_ decays to zero almost instantaneously, compared with the slower term associated with τ_A_.

Therefore, a dominant pole approximation can be considered for G_2_(s) and Equation (14) can be rearranged as

(15) $$G_{2}\left(s\right)≅\frac{1}{R_{s}}\cdot \frac{\frac{1}{\tau_{\mathrm{in}}\cdot \tau_{A}}}{\left(s+\frac{1}{\tau_{\mathrm{in}}}\right)\left(s+\frac{1}{\tau_{A}}\right)}≅\frac{1}{R_{s}}\cdot \frac{1}{\left(1+\tau_{A}s\right)}.$$

Furthermore, at high frequencies, which describe well the leading edge of the output pulse we are interested in, the following assumption can be made:

$$\frac{1+\tau_{q}s}{1+\tau_{p}s}≅\frac{\tau_{q}}{\tau_{p}}=\alpha ,$$

leading to further simplification of Equation (12), which becomes

(16) $$V_{I,\mathrm{out}}\left(s\right)≅\alpha \cdot V_{0}\cdot \frac{1}{R_{s}}\cdot \frac{1}{s}\cdot \frac{1}{\left(1+\tau_{A}s\right)}\cdot \frac{K_{R}}{\left(1+\tau_{L}s\right)}$$

The Laplace transform of the slope of the output pulse, ${\mathrm{Slope}}_{V,I}\left(s\right)$, is obtained by multiplying expression (16) by the variable s:

(17) SlopeV,I(s)=α·V0·KRRs·1τAτL·(as+1τA+bs+1τL),

where $a=-\frac{\tau_{A}\cdot \tau_{L}}{\tau_{L}-\tau_{A}}$ and $b=\frac{\tau_{A}\cdot \tau_{L}}{\tau_{L}-\tau_{A}}$.

Equation (17) can be conveniently used to derive simple expressions for both the time corresponding to the maximum slope, $t_{{\mathrm{MAX}}_{S}}$, and the value of the maximum slope of the output pulse as a function of the most relevant parameters involved.

The inverse Laplace transform of Equation (17) leads to the final expression for the slope of the output voltage signal in the time domain:

(18) $${\mathrm{Slope}}_{V,I}\left(t\right)={ℒ}^{-1}\left\{{\mathrm{Slope}}_{V,I}\left(s\right)\right\}=\alpha \cdot V_{0}\cdot \frac{K_{R}}{R_{s}}\cdot \frac{1}{\tau_{A}\cdot \tau_{L}}\cdot \left(a\cdot e^{-\frac{t}{\tau_{A}}}+b\cdot e^{-\frac{t}{\tau_{L}}}\right).$$

Taking the time derivative of ${\mathrm{Slope}}_{V,I}\left(t\right)$, equating it to zero and solving for the time variable t, the time $t_{{\mathrm{MAX}}_{S}}$, expressed as a function of τ_A_, τ_L_, L_par_, and R_s_ is

(19) $$t_{{\mathrm{MAX}}_{S}}=\frac{\tau_{A}\cdot \tau_{L}}{\tau_{A}-\tau_{L}}\cdot \ln\left(\frac{\tau_{A}}{\tau_{L}}\right)=\frac{\tau_{A}}{\theta -1}\cdot \ln\theta ,$$

where θ=τAτL is a normalization variable, depending on $\tau_{A}=L_{\mathrm{par}}/R_{S}$ and $\tau_{L}$. The maximum slope, i.e., Equation (18) evaluated for $t=t_{{\mathrm{MAX}}_{S}}$, is the following:

(20) $${\mathrm{Slope}}_{V,I}\left(t_{{\mathrm{MAX}}_{S}}\right)=\alpha \cdot V_{0}\cdot \frac{K_{R}}{R_{s}}\cdot \frac{1}{\tau_{A}}\cdot e^{-\frac{t_{{\mathrm{MAX}}_{S}}}{\tau_{A}}},$$

and, replacing $t_{{\mathrm{MAX}}_{S}}$ with Equation (19) and the transimpedance gain $K_{R}$ with the product $A_{i}\cdot R_{L}$, the following expression results:

(21) $${\mathrm{Slope}}_{V,I}\left(t_{{\mathrm{MAX}}_{S}}\right)=\alpha \cdot V_{0}\cdot \frac{A_{i}\cdot R_{L}}{L_{\mathrm{par}}}\cdot \theta^{\frac{1}{1-\theta }}.$$

The corresponding expression for the voltage-mode approach is

(22) $${\mathrm{Slope}}_{V,V}\left(t_{{\mathrm{MAX}}_{S}}\right)=\alpha \cdot V_{0}\cdot \frac{A_{v}\cdot R_{\mathrm{in}}}{L_{\mathrm{par}}}\cdot \theta^{\frac{1}{1-\theta }},$$

where the dominant time constant τ_AMP_ of the voltage amplifier replaces τ_L_ in the parameter θ.

Equations (21) and (22) are very simple expression which describe the dependence of the maximum slope of the output pulse on the parameter θ, which, in turns, depends on the time constants $\tau_{A}$, i.e., the ratio $L_{\mathrm{par}}/R_{S}$, and the dominant time constant of the preamplifier.

It is apparent that the maximum slopes obtained in case of application of a current-mode or voltage-mode front-end approach share the same dependence on the parameter θ. However, Equation (22) exhibits also an explicit dependence of the maximum slope on the input resistance of the preamplifier R_in_. Consequently, if a voltage-mode approach is used, the maximum slope of the output pulse tends to increase when the input resistance increases, whereas the opposite happens in case the current-mode approach is adopted.

**Figure 8:**
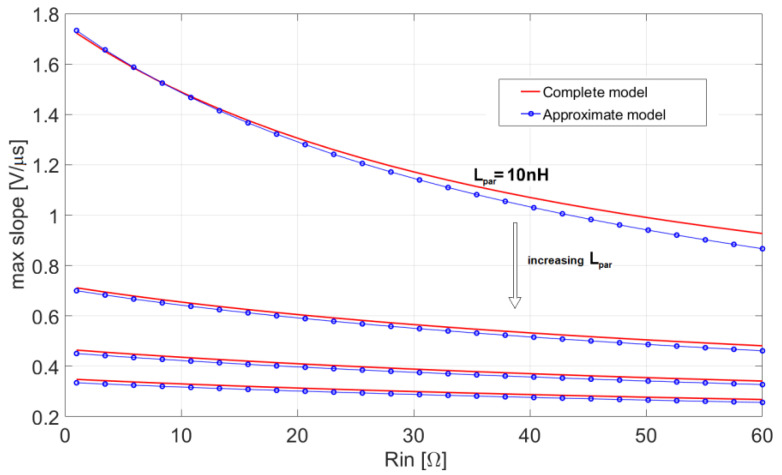
Maximum slope of the leading edge of the output pulse for the current-mode approach (12), compared with its approximation (21), with L_par_ = 10 nH, 40 nH, 70 nH, 100 nH, C_in_ = 0.5 pF and BW = 0.5 GHz.

**Figure 9:**
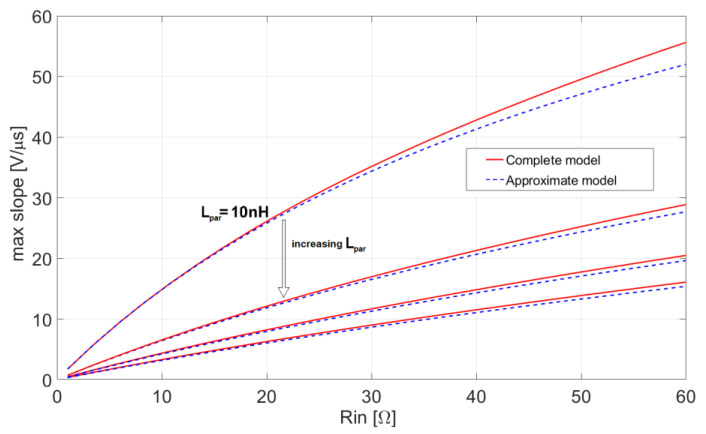
Maximum slope of the leading edge of the output pulse for the voltage-mode approach (13) compared with its approximation (22), with L_par_ = 10 nH, 40 nH, 70 nH, 100 nH, C_in_ = 0.5 pF, and BW = 0.5 GHz.

These conclusions are illustrated by the following Figure 8 and Figure 9, which compare the exact slopes, obtained using the complete models (12) and (13), with their corresponding approximations (21) and (22), respectively. In both Figure 8 and Figure 9 the maximum slope is represented as a function of R_in_ for four values of the inductance L_par_. The plots prove that the proposed lower order approximation of the complete system gives very accurate results.

**Figure 10:**
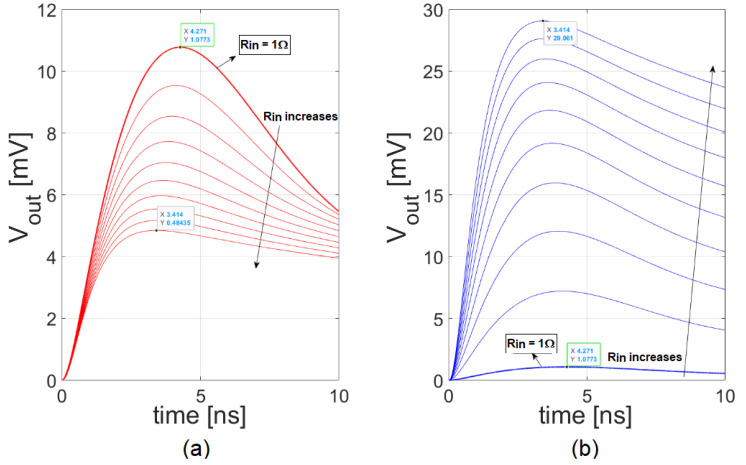
The current-mode (**a**) and voltage-mode (**b**) responses as functions of the input resistance when L_par_ = 70 nH, C_in_ = 0.5 pF, and BW = 0.5 GHz, obtained using the complete model.

The same conclusions can be drawn observing the plots in Figure 10. Here, the output waveforms resulting from both a current-mode approach and a voltage-mode approach, obtained with the complete model, are shown for different values of R_in_. As R_in_ increases, the system becomes faster for both the current-mode and the voltage-mode, thanks to the decreasing value of the time constant τ_A_ = L_par_/(R_in_ + R_par_). This causes the decrease of the peaking time observed in both graphs of Figure 10 for increasing values of R_in_. However, in the current-mode case, increasing the input resistance causes also a decrease of the amplitude of the current pulse I_in_(t) and, consequently, of the output voltage pulse. This results in a net reduction of the maximum output slope, despite the decrease of the peaking time. Instead, in the voltage-mode case, the increase of R_in_ also causes an increase of the amplitude of the output pulse and, consequently, of its maximum slope.

**Figure 11:**
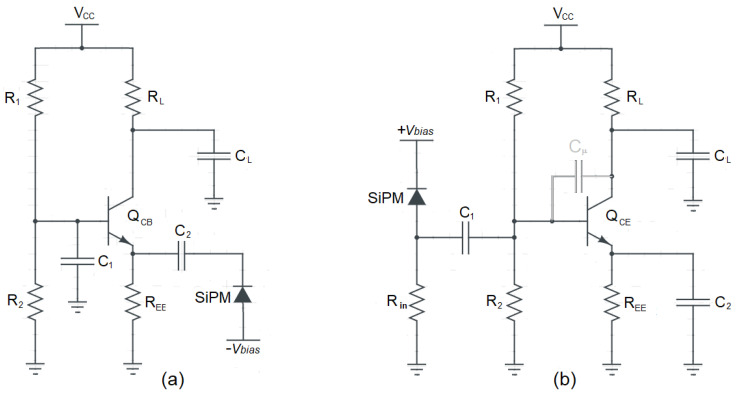
Two readout approaches: (**a**) a BJT in the common base configuration; (**b**) the same BJT in the common emitter configuration.

A clear advantage of the current-mode approach is that, owing to the absence of internal high impedance nodes, it is preferable compared to a voltage-mode approach in case large bandwidth is needed. Let us consider, for instance, a simple current buffer realized by means of the common base (CB) amplifier depicted in Figure 11a. The transimpedance gain of the circuit is given by the load resistance R_L_, whereas the time constant associated to the load capacitance τ_L_ = R_L_C_L_ determines its bandwidth, according to the system shown in Figure 1. Moreover, a basic voltage-mode implementation of the front-end using the same active device is the common-emitter (CE) amplifier shown in Figure 11b.

If the open-circuit time constant method is applied to estimate the dominant time constant $\tau_{\mathrm{AMP}}$ of the CE amplifier, we obtain

(23) $$\tau_{\mathrm{AMP}}≅R_{L}C_{L}+\left(R_{\mathrm{in}}||r_{\pi }\right)C_{\pi }+R_{\mathrm{in}}C_{\mu }A_{V},$$

where $A_{V}=g_{m}{}_{\mathrm{CE}}R_{L}.$

To make the bandwidth of the CE equal to the bandwidth of the current buffer, both the second and the third terms in Equation (23) should be negligible. Under the assumption that the input resistance is small enough to make the second term very small, by imposing

(24) $$R_{\mathrm{in}}C_{\mu }g_{m}{}_{\mathrm{CE}}R_{L}\ll R_{L}C_{L}$$

the following condition on the value of R_in_ is obtained:

(25) $$R_{\mathrm{in}}\ll \frac{1}{g_{m}{}_{\mathrm{CE}}}\cdot \frac{C_{L}}{C_{\mu }}.$$

Condition (25) states that, in practice, in this example of voltage-mode circuit it is impossible to exploit the increase of the input resistance to increase the slope of the output pulse. In fact, this would result in an unavoidable penalty in terms of bandwidth, in comparison to the corresponding current-mode circuit. Increasing the transconductance would have a beneficial effect on the voltage gain of the circuit, thus on the response slope. However, this would also require a further decrease of the input resistance to fulfil the condition (25); otherwise, the bandwidth would be compromised, thus neutralizing the benefits in terms of slope. Instead, in the CB case, increasing the transconductance, and hence the power consumption, would decrease the input resistance of the current-mode circuit, thus increasing the slope of the output pulse, without affecting the bandwidth of the preamplifier.

**Figure 12:**
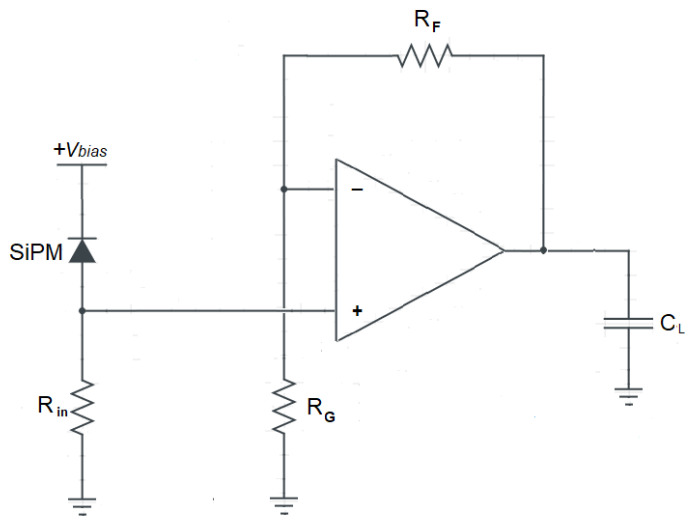
A voltage-mode preamplifier, based on an op-amp.

Another possible example of voltage-mode front-end is reported in Figure 12, based on a simple op-amp non-inverting configuration.

In this case, the closed-loop bandwidth is expressed as

(26) $$\mathrm{BW}=\frac{\mathrm{GBW}}{A_{V}},$$

where GBW is the gain-bandwidth product of the op-amp, and $A_{V}=1+R_{F}/R_{G}$ is the DC closed-loop voltage gain.

Referring to Equations (21) and (22), the maximum slopes obtained with the two front-end approaches are the same if $A_{V}R_{\mathrm{in}}=A_{I}R_{L}$ and also if BW = 1/2πτ_L_ = 1/2πR_L_C_L_. Therefore, we must have

(27) $$R_{\mathrm{in}}=\frac{A_{I}R_{L}}{A_{V}}=\frac{A_{I}R_{L}}{\mathrm{GBW}}\cdot \mathrm{BW}=\frac{A_{I}}{2\pi \cdot \mathrm{GBW}\cdot C_{L}}.$$

Equation (27) states that if we want to obtain advantages in terms of maximum slope of the output signal with the voltage-mode circuit in Figure 12, with respect to the current-mode circuit in Figure 11a, its input resistance R_in_ must be very large. In fact, since C_L_ is of the order of few pF, to keep the value of R_in_ provided by Equation (27) within reasonable limits, i.e., around few hundreds of Ohms, huge values of GBW would be needed. If R_in_ is increased beyond these limits, the time constant τ_in_ cannot be considered negligible any longer, slowing down the rise time of the output pulse, due to the contribution of this further time constant. Moreover, with large values of R_in_, the duration of the long tail of the output pulse increases, causing pile-up problems and worsening the timing accuracy of the circuit, due to baseline fluctuations [25,26]. In conclusion, also for this further example of voltage-mode preamplifier, exploiting the increase of the input resistance R_in_ to improve the timing performance of the detection system is not practically feasible.

## 5. Experimental Tests and Results

**Figure 13:**
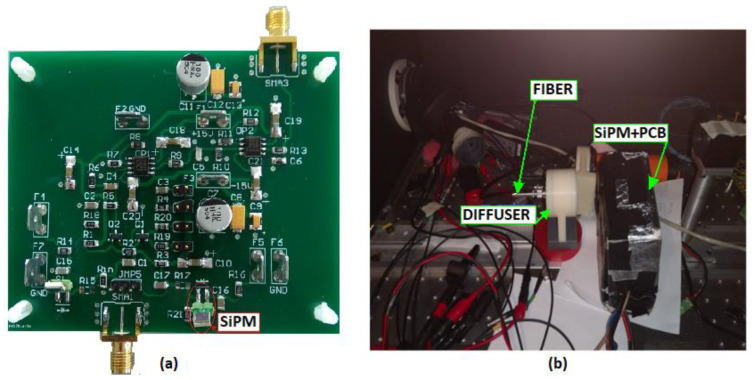
Pictures of (**a**) the printed circuit board used for the experiments and (**b**) the experimental setup in the dark box.

To confirm the validity of the proposed analysis with experimental results, a printed circuit board (PCB) has been designed to readout the response of the S10931-050P SiPM from Hamamatsu (see Table 1). In this board, a fast RF BJT configured as a common base current buffer, as shown in Figure 11a, is used in the very front-end. Figure 13 shows the PCB with the 3 × 3 mm^2^ SiPM and the experimental setup, enclosed in dark box.

The SiPM can be coupled to the input of the preamplifier directly, using a zero-ohm resistor, or by interposing a small discrete inductor with two possible values, L = 51 nH or L = 100 nH. Moreover, four possible different values of R_in_ can be selected, i.e., 10 Ω, 18 Ω, 33 Ω, and 50 Ω, by changing the emitter resistor R_EE_ that sets the DC current of the transistor, as shown in Figure 11. A large-bandwidth voltage amplifier, cascaded to the front-end, has been used to increase the amplitude of its output pulse. A 380 nm fast pulsed laser source has been used to generate light flashes with FWHM duration of 50 ps. The light is sent towards the SiPM sensitive surface by means of an optical fiber and an optical diffuser has been interposed between the fiber and the detector, to maximize the probability of the detection of single-photon events.

**Figure 14:**
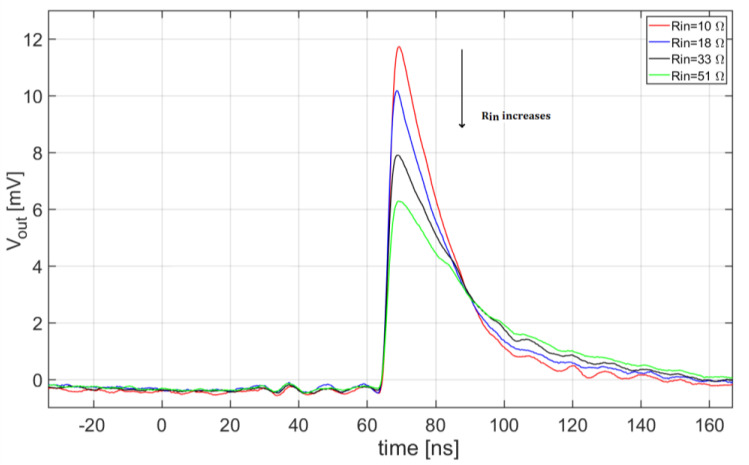
Examples of ‘golden’ pulse waveforms, obtained with L = 51 nH and different values of R_in_.

Among all the responses of the circuit to the laser flashes, acquired in coincidence with the laser trigger, only the ones corresponding to single-photon events have been selected. For each possible circuit arrangement in terms of input resistance and series inductance, a noiseless ‘golden’ waveform has been extracted, by aligning and averaging the acquired pulses. Examples of such waveforms obtained with L = 51 nH and different values of R_in_ are reported in Figure 14.

**Figure 15:**
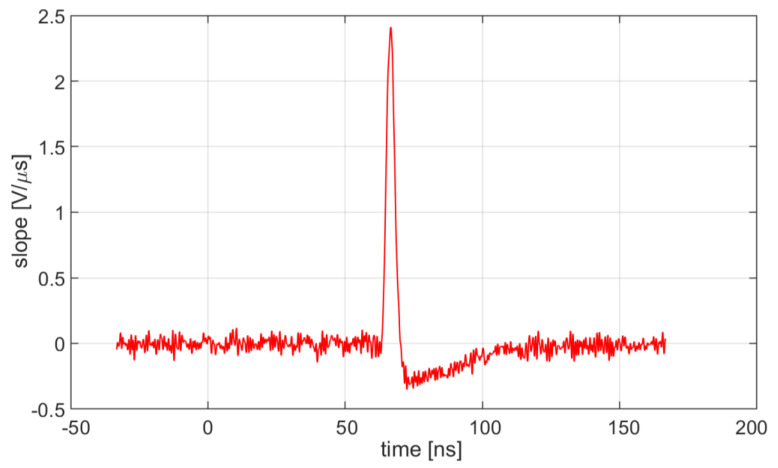
An example of time derivative of a ‘golden’ pulse, obtained with L = 51 nH and Rin = 33 Ω.

The derivative of each ‘golden’ pulse and its maximum value have been evaluated. Figure 15 shows an example of such derivative, obtained with L = 51 nH and R_in_ = 33 Ω.

**Figure 16:**
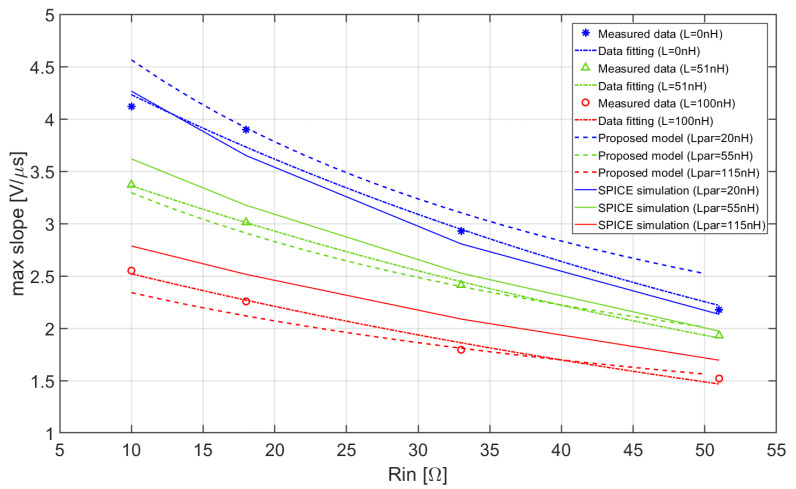
Maximum slopes of the output pulse of the front-end as a function of the input resistance R_in_ for three values of parasitic inductance: comparison among measured data, proposed model predictions, and SPICE simulations.

Finally, the peak values of the slopes have been plotted as a function of the input resistance for the three possible values of series inductance, as shown in Figure 16. The fitting curves of the experimental data and the corresponding behavior of the maximum slope obtained respectively by means of the proposed mathematical model and by SPICE simulations of the circuit are also reported in Figure 16.

It is apparent from Figure 16 that the behavior of the measured data basically reproduces the one predicted by the approximate analytical model and by the SPICE simulations. Small corrections of the series inductance have been introduced in the model with respect to the ideal value of the inductance L. These corrections are needed to take account of the unavoidable parasitic contributions associated to the zero-ohm resistor and the interconnections, slightly variable when a different physical inductor is interposed between the SiPM and the front-end. As also shown in Figure 16, these corrections increase the total value of the inductance L_par_ to 20 nH, 55 nH, and 115 nH, respectively in case the zero-ohm resistor, the 51 nH and the 100 nH inductors are inserted. Whatever the value of the series inductance, when the input resistance R_in_ of the CB amplifier is increased, the maximum slope decreases, as predicted by the proposed model.

**Figure 17:**
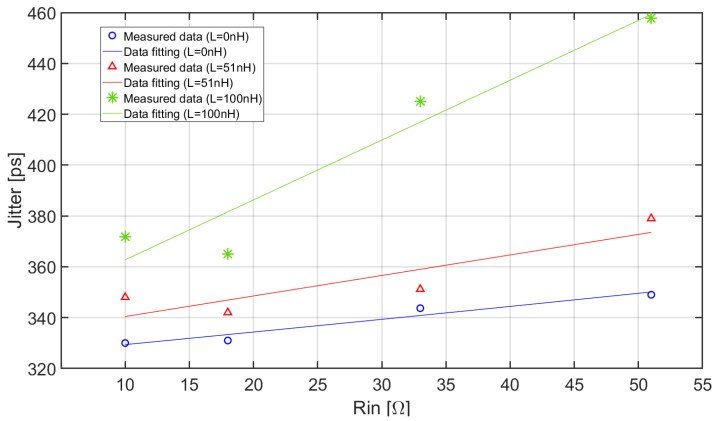
Time jitter as a function of R_in_ and L. The light continuous curves are first order exponential fittings of the measured data points.

Figure 17 shows the time jitter as a function of the input resistance for the three different values of series inductance.

The jitter has been evaluated using the time spread distribution of the delay between the trigger pulse of the laser and the time when the output signal resulting from the detection of a single photo-electron event reaches the threshold. The threshold has been fixed at the level corresponding to the maximum slope of the ‘golden’ pulse. The curves in Figure 17 include the contributions of the measurement setup to the total jitter.

Considering low and moderate values of the series inductance, the jitter is poorly dependent on the input resistance and slightly increases when R_in_ increases. This behavior is consistent with both experimental data reported in Figure 16 and Equation (21), since the slope decreases when R_in_ increases and, in our circuit, the electronic noise is dominated by the contribution associated with the resistor R_L_ (see Figure 11), thus it does not change significantly. With higher inductance values (green curve), the time resolution rapidly degrades when R_in_ increases. This suggests that, when a current-mode approach is used, choosing low values for the input resistance of the front-end electronics has relevant advantages in terms of low input jitter especially when the parasitic interconnection inductance is relatively large. Conversely, when the SiPM is coupled directly to the front-end with very short interconnections and L_par_ is very small, the influence of R_in_ on the timing performance of the circuit is, to some extent, reduced. In this case, the advantages of the decrease of R_in_ must be compared to possible drawbacks, such as, for instance, an increase of the power consumption of the circuit.

## 6. Conclusions

Analytical expressions for the single-photon response of a SiPM-based detection system are already available in the literature. However, these models either do not account for relevant factors, such as the parasitic inductance interposed between the detector and the front-end electronics, or are based on inaccurate electric models of the SiPM.

Starting from a complete model of the detector and considering reasonable assumptions, we propose a new factorized expression of the transfer function of the system, able to accurately reproduce its behavior in the initial transient of the response. In this expression, the individual contributions of the SiPM, the interconnection parasitic components, and the front-end electronics have been distinguished as independent blocks.

Based on this model, a simple but accurate closed-form equation for the single-photon response as a function of the main system parameters has been derived. As is well known, the slope of the response has a relevant influence on the timing performance of the system, when leading edge discrimination is the chosen time pickoff technique. Our simplified model reproduces with good accuracy the slope of the initial transient of the response provided by the complete model of the system, when its parameters are varied.

The proposed analysis allows to easily perform a direct comparison of the single-photon response obtained with different front-end architectures. The results of this comparison show that a current-mode readout approach is preferable to a voltage-mode one in terms of timing accuracy, with typical values of the parameters involved.

Moreover, practical design guidelines for the front-end electronics can be devised by means of the proposed analytical model of the response. For instance, when a current-mode approach is chosen, we found that extremely low values of the input resistance of the preamplifier are particularly convenient only in presence of increasing values of the parasitic interconnection inductance.

Last, experimental tests, carried out on an example of current-mode front-end circuit coupled to a 3 × 3 mm^2^ SiPM, have been used to validate the results of our analysis. The behavior of the real readout system has been reproduced with good accuracy by our approximate model, in terms of maximum slope of the single-photon response and resulting time jitter.

## Figures and Tables

**Table 1:** Electrical parameters of the SiPM used in the simulations.

**R_q_**	182.75 kΩ
**C_q_**	17.72 fF
**C_d_**	75.17 fF
**C_g_**	36.85 pF
**R_sub_**	22.9 Ω
**N**	3600
